# Determinants of tuberculosis disease development in children in central Ethiopia: A matched case-control study

**DOI:** 10.1371/journal.pone.0300731

**Published:** 2024-05-09

**Authors:** Abay Burusie, Fikre Enquesilassie, Nicole Salazar-Austin, Adamu Addissie

**Affiliations:** 1 Department of Public Health, College of Health Sciences, Arsi University, Asella, Ethiopia; 2 School of Public Health, College of Health Sciences, Addis Ababa University, Addis Ababa, Ethiopia; 3 Division of Pediatric Infectious Diseases, Johns Hopkins University School of Medicine, Baltimore, Maryland, United States of America; Hawassa University College of Medicine and Health Sciences, ETHIOPIA

## Abstract

**Background:**

The risk factors for tuberculosis (TB) disease development in children remained understudied, particularly in low-income countries like Ethiopia. The objective of this study was to identify determinants of TB disease development in general and in relation to BCG vaccination in children in central Ethiopia.

**Methods:**

We employed a 1:1 age-matched case-control design to compare the characteristics of children who developed TB (cases) with those who did not (controls). Data were collected in healthcare facilities in Addis Ababa city, Adama, and Bishoftu towns between September 25, 2021, and June 24, 2022. Two hundred and fifty-six cases were drawn at random from a list of childhood TB patients entered into SPSS software, and 256 controls were selected sequentially at triage from the same healthcare facilities where the cases were treated. A bivariate conditional logistic regression analysis was performed first to select candidate variables with p-values less than or equal to 0.20 for the multivariable model. Finally, variables with a p-value less than 0.05 for a matched adjusted odds ratio (mORadj) were reported as independent determinants of TB disease development.

**Results:**

The mean age of the cases was nine years, while that of the controls was 10 years. Males comprised 126 cases (49.2%) and 119 controls (46.5%), with the remainder being females. Ninety-nine (38.7%) of the cases were not BCG-vaccinated, compared to 58 (22.7%) of the controls. Household TB contact was experienced by 43 (16.8%) of the cases and 10 (3.9%) of the controls. Twenty-two (8.6%) of the cases and six (2.3%) of the controls were exposed to a cigarette smoker in their household. Twenty-two (8.6%) of the cases and three (1.2%) of the controls were positive for HIV. Children who were not vaccinated with BCG at birth or within two weeks of birth had more than twice the odds (mORadj = 2.11, 95% CI = 1.28–3.48) of developing TB compared to those who were. Children who ever lived with a TB-sick family member (mORadj = 4.28, 95% CI = 1.95–9.39), smoking family members (mORadj = 3.15, 95% CI = 1.07–9.27), and HIV-infected children (mORadj = 8.71, 95% CI = 1.96–38.66) also had higher odds of developing TB disease than their counterparts.

**Conclusions:**

Being BCG-unvaccinated, having household TB contact, having a smoker in the household, and being HIV-infected were found to be independent determinants of TB disease development among children.

## Introduction

Mycobacterium tuberculosis (MTB) is estimated to infect nearly a quarter of the global population [1]. More than 90% of TB disease in children under the age of 15 develops within a year of infection [2].

Tuberculosis (TB)-infected children under the age of one year and under the age of five have the same chance of developing TB (18% and 19%, respectively) [3]. Children under the age of five, however, have a significantly higher rate of progression to TB disease than older children, such as those aged 5 to 9 years, 10 to 14 years, and 15 to 18 years [3].

Other than age, a number of factors have been found to increase the risk of latent TB infection developing into TB disease, which include: close contact with a TB patient [4–6]; living in a crowded household [4–6]; chronic illnesses such as HIV infection [4–6], diabetes mellitus (DM) [7–9], end-stage renal disease, liver cirrhosis, and chronic obstructive pulmonary diseases [7]; immune-compromising conditions including malignancy, steroid use, and undernutrition [6, 10, 11].

MTB strain type is also important in the development of TB disease because it influences the innate immune response [12]. The East Asian/Beijing genotype was independently associated with a shorter duration of illness before presentation compared to the Euro-American genotype [13].

Some studies have also identified passive cigarette smoking as a risk factor for TB in children [10, 14], but we lack studies that evaluate passive smoking as a risk factor for TB in Ethiopian children [15, 16].

The BCG vaccine, developed in 1921 from live attenuated Mycobacterium bovis, is known to be protective against disseminated TB disease in young children. However, there is still some disagreement about how long the BCG vaccination is effective against TB. Some studies have shown that BCG vaccination is protective against TB for 10–20 years post-vaccination, after which the effect fades [17–19]. A recent meta-analysis demonstrated that BCG only significantly protects against TB disease in children under the age of five, not in adolescents or adults [20]. Furthermore, studies reveal that the BCG vaccine is less effective in protecting against TB near the equator, which includes Ethiopia, highlighting the need for additional studies in the region to corroborate the findings [21, 22].

The WHO-notified BCG strains include the French Pasteur strain 1173 P2, the Danish strain 1331, the Glaxo strain 1077, and the Tokyo strain 172, which together account for about 90% of BCG vaccinations worldwide [23]. No BCG strain is demonstrably better than another in efficacy, and there is no global consensus as to which strain of BCG is optimal for general use [23, 24]. Ethiopia administers the Danish strain [25].

Ethiopia has a long-standing and up-to-date National Implementation Guideline for Expanded Immunization Program that outlines the roles and duties of various stakeholders involved in vaccine supply, cold chain, and quality control [26] in accordance with WHO standards [27]. As a result, the Ministry of Health guarantees that potent vaccines are available at all levels. The Ethiopian Food and Drug Authority (FDA) is in charge of licensing WHO pre-qualified vaccinations, as well as monitoring and managing adverse occurrences. The Ethiopian Pharmaceutical and Supply Agency (EPSA) distributes vaccinations for health facilities while maintaining vaccine quality through cold chain distribution. All health facilities and stores that handle vaccines are regularly trained and supervised to use standard refrigerators (WHO PQS prequalified) to store the vaccines and must monitor and record the temperature of the vaccines in the cold chain at least twice daily, including weekends and holidays, to ensure timely action [26].

The fact that risk factors for TB disease development in children such as exposure to a household smoker have been inadequately investigated in low-income countries such as Ethiopia, as well as the ongoing debate over how long BCG protects against TB, highlights the need for additional research. We aimed to identify determinants of TB disease development in general and in relation to BCG vaccination in children in central Ethiopia.

## Methods

### Study design

We employed a 1:1 matched case-control design to compare the characteristics of children who developed TB disease (cases) to those who did not (controls).

### Study setting and period

The study was conducted in healthcare facilities in Addis Ababa city as well as Adama, and Bishoftu towns in central Ethiopia. Adama and Bishoftu are larger urban areas located within a 100-kilometer radius of Addis Ababa, Ethiopia’s capital. Residents here had easy access to telephones to be accessed for phone interviews. Being closer to Addis Ababa helps patients have readily accessible access to advanced diagnostic procedures, reducing the possibility of TB misdiagnosis.

Data were collected between June 01, 2022, and June 24, 2022, among the non-TB control study participants. Data on TB cases were obtained from TB register books and via phone interview, mostly from September 25, 2021, to December 28, 2021, not only for the purposes of this study but also to do a survival analysis of children treated for TB. Furthermore, we waited for certain cases until they finished their TB treatment or their treatment outcome was known before resuming collecting data on them from May 10, 2022, to June 15, 2022, depending on the date the treatment outcome for one was known and recorded on the TB treatment register.

### Cases

The cases were children aged 16 and under who had been diagnosed with pulmonary TB (PTB) or extra-pulmonary TB (EPTB) and treated with TB drugs. The patients that were treated for TB and whose outcomes were known between May 1, 2015, and June 15, 2022, were extracted from the TB registers.

### Controls

The controls were children who were born the same year as the matching cases and treated for non-TB conditions, or received health promotion or preventive services between June 1, 2022, and June 24, 2022 in the same healthcare facilities where the cases were treated for TB. The controls were individuals aged 18 and under who had never been diagnosed with PTB or EPTB based on verbal reports from their parents or caregivers. The controls were chosen from the same birth cohort as the cases, with the cases’ age at the time of TB diagnosis used to estimate the age of our controls during the interview. Furthermore, controls were screened to ensure they were currently free of potential TB symptoms listed in the WHO [28] and Ethiopia’s TB national guidelines [29], such as prolonged cough for ≥ 2 weeks, fever, night sweets, weight loss, and extra-pulmonary site or organ-specific TB symptoms such as cervical or axillary lymphadenopathies, spinal deformity (kyphosis or gibbus), and signs of meningeal irritation (neck stiffness, seizure).

### Matching

Child TB patients were matched 1:1 on age and study facilities with children who had never experienced TB based. Childhood TB is difficult to diagnose and is commonly delayed [30].To avoid wrongly enrolling such unrecognized early-stage active TB or sub-clinical (asymptomatic but infectious) TB patients as controls [31], the controls were recruited after the cases’ TB treatment outcomes were determined. Delaying control recruitment allow for more time for an unrecognized or subclinical TB to manifest clearly, lowering the risk of misclassification bias. Age was chosen as a matching variable with the premise that it is a strong confounder.

### Study variables

The dependent variable was having ever had TB disease. The primary independent variable was BCG vaccination at birth or within two weeks after birth. Sex, HIV status, a cigarette-smoking family member living with the child, and prior or concurrent household TB patient contact were considered control variables.

### Sample size determination

A formula to determine sample size for individually matched studies with 1:1 matching [32] was used to compute the required number of cases. Because BCG scar is the sensitive marker of BCG vaccination [33], we used its proportion in the population to calculate our sample size. Taking Z_α/2_ of 1.96 for 5% significance level (α), Z_β_ of 0.84 for 80% power, and assuming 81% of children are exposed to BCG scar [34], the required number of cases to detect odds ratio of 2.00 was calculated to be 256. As a result, the total number of cases and controls became 512, yielding 256 matched case-control pairs.

### Sampling technique and data collection

The sampling frame for the random selection of cases was a list of 524 childhood TB patients entered into SPSS software and whose parents or caregivers were interviewed by phone during another study with a different objective than this one (the specifics of how and how many facilities were chosen are detailed in Burusie A. et al. [35]). Controls were selected sequentially at triage from the same healthcare facilities where the cases were treated.

We collected data from the controls using a face-to-face interviewer-administered questionnaire. We used a checklist to extract data on the cases from their TB treatment registration books. We collected the data that could not be collected using the checklist from the cases—the information that did not have a space to fill in the TB register—by conducting a telephone interview with parents or caregivers. The following information was collected by phone interview because it did not fit into the TB registry: BCG vaccination status, child’s schooling, the existence of a smoking family member living with the TB-sick child, and the presence of a TB patient in the household. Variables such as child’s gender, age, nutritional status, HIV status, and household size were obtained from the patient’s TB register.

The data were collected by clinical nurses and health officers.

### Data analysis

We used EpiData version 3.1 to enter the data. The data was then imported into Stata version 14 for further cleaning and analysis. A conditional logistic regression model was used to assess the association using a matched crude odds ratio (mCOR) with a 95% confidence interval (CI). Variables that attained p-values less than or equal to 0.2 during the bivariate analysis were chosen as candidates for the multivariable analysis. Finally, variables with a p-value less than 0.05 for the matched adjusted odds ratio (mORadj) were reported as significant determinants of TB disease. The odds ratios were calculated from discordant pairings. We used the STROBE case-control checklist when writing our report [36].

### Ethics statement

The Institutional Review Board (IRB) of Addis Ababa University’s College of Health Sciences (protocol number: 057/19/SPH) approved the study. The ethical approval was originally obtained from the IRB, which was good for the period of October 22, 2020, to October 21, 2021. The ethical clearance was then renewed for another year, from May 19, 2022, to May 18, 2023.

We obtained letters of support from the Addis Ababa City Administration’s and Oromia Regional State’s Health Bureaus for the study’s healthcare facilities. Permission to access the TB registration and contact patients’ parents or caregivers was then obtained from the medical directors of the study healthcare facilities. Parents or guardians of TB-sick children (cases) gave oral consent before a phone interview was conducted. Because the interviews for the cases were conducted over the phone to ask questions such as BCG vaccination status, household TB contact, exposure to household smokers, and educational level, which were not recorded on the TB patients follow-up register book, obtaining written consent was impractical. The oral consents were documented by field notes on response and non-response counts and witnessed by the primary investigator phoning the parents as part of a data quality check.

Although it was possible to obtain written consent from controls to participate in the interview, it was a time of great instability in Ethiopia due to the civil war, and citizens used to see things with a high level of suspicion in spite of being given information on the purpose of the study, confidentiality, and the fact that their data would not be used for any other purpose than identifying predisposing factors for childhood TB and being assured of the right to refuse to participate. As a result, we were compelled to obtain oral consent because they were afraid and worried about placing their signature on a consent form. The IRB approved the oral consent.

Only the principal investigator had access to information that could identify individual participants after data collection for data quality check purposes by calling parents or guardians.

## Results

### Characteristics of childhood TB patients and the controls

We identified and analyzed 256 TB patients (cases) and 256 people who had never had TB (controls). Sputum smear microscopy and X-ray were used to diagnose 63 (24.6%) of TB patients, while GeneXpert/RIF was used to diagnose 52 (20.3%) of them. The remaining 141 (55.1%) patients were clinically diagnosed with a high index of suspicion and TB-suggestive imaging.

Males comprised 126 cases (49.2%) and 119 controls (46.5%), with the remainder being females. The cases had a mean age of nine years, while the controls had a mean age of ten years. One hundred eighty (70.3%) of the cases and 180 (70.3%) controls were chosen from Addis Ababa. Some settlements that were previously part of Addis Ababa were relocated to the Oromia region under the new demarcation, and vice versa. However, they continue to get health care services from the neighboring Addis Ababa-based health facility or Oromia-based health facility where they were previously served, resulting in a disparity in the number of cases and controls selected from the regions. Ninety-nine (38.7%) of the cases were unvaccinated with BCG, compared to 58 (22.7%) of the controls. Forty-three (16.8%) cases were exposed to household TB contact, whereas only 10 (3.9%) of the controls were. Twenty-two (8.6%) of the cases and six (2.3%) of the controls had a cigarette smoker in their households. Twenty-two (8.6%) of the cases and three (1.2%) of the controls were HIV-infected. The largest household size among the cases was 30, which came from a foster center where numerous children lived in the same room, whereas it was 9 among the controls (Table 1).

**Table 1:** Characteristics of cases at the time of TB diagnosis and that of controls at time of survey in a study conducted in central Ethiopia, 2022.

Characteristics	Category	Cases (%) N = 256	Controls (%) N = 256
**Sex**	Male	126 (49.2)	119 (46.5)
	Female	130 (50.8)	137 (53.5)
**Age in years at TB diagnosis for cases and at survey time for controls**	Mean (standard deviation); median	8.96(5.5); 10	10.3(5.3); 10
**Study site**	Addis Ababa city	180 (70.3)	180 (70.3)
	Adama town	26 (10.2)	26 (10.2)
	Bishoftu town	50 (19.5)	50 (19.5)
**Nutritional status**	Normal	176 (68.8)	248 (96.9)
	Undernourished	80 (31.2)	8(3.1)
**BCG vaccination within two weeks of birth**	Yes	157 (61.3)	198 (77.3)
	No	99 (38.7)	58 (22.7)
**Child’s Education**	Kindergarten	23 (13.2)	40 (19.6)
	Grade 1–6	98 (56.3)	86 (42.2)
	Grade 7–8	34 (19.5)	38 (18.6)
	Grade 9–12	19 (10.9)	40 (19.6)
**Household family member sick with TB**	Yes	43 (16.8)	10 (3.9)
	No	213 (83.2)	246 (96.1)
**Cigarette smoking family member**	Yes	22 (8.6)	6 (2.3)
	No	234 (91.4)	250 (97.7)
**HIV status**	Positive	22 (8.6)	3 (1.2)
	Negative	234 (91.4)	61 (23.8)
	Unknown	0 (0)	192(75.0)
**Household size**	Minimum; Maximum; mean; median	2; 30; 5.2; 4	2;9; 4.7; 5

### Determinants of TB disease in children

We analyzed 256 case-control pairings in total. Sixty-six (25.8%) of case-control pairings were discordant in the sense that the cases were not vaccinated for BCG while the controls were, and 25 (9.8%) of the pairings were discordant in the sense that the cases were vaccinated while the controls were not. The cases had household TB contact while the controls did not in 42 (16.4%) discordant pairings, and the cases had no contact while the controls did in 9 (3.5%) discordant pairings. With regard to cigarette smoking, discordant pairings included 21 (8.2%) cases that had ever lived with a smoking family member but the controls had not, and 5 (2.0%) cases that had never lived with a smoking family member but the controls had. There were 21 (8.2%) discordant pairings when cases were HIV-positive and controls were not and 2 (0.8%) discordant pairings when controls were HIV-positive and cases were not. In the multivariable analysis, the odds of being unvaccinated for BCG at birth or within two weeks after birth was found to be more than twice as high (mORadj = 2.11, 95% CI = 1.28–3.48) among TB patients as in the never-had-TB groups. Our study also found that the odds of developing TB disease among those children who had household TB patient contact was significantly higher than four times the odds of developing TB disease among those who did not have household TB contact (mORadj = 4.28, 95% CI = 1.95–9.39). Likewise, children who lived with a smoking family member had significantly higher odds of developing TB than children who did not (mORadj = 3.15, 95% CI = 1.07–9.27). Furthermore, TB patients had more than eightfold higher odds of HIV infection than non-TB groups (mORadj = 8.71, 95% CI = 1.96–38.66). However, household size (as a continuous covariate) had no statistically significant association with the development of TB disease (mORadj = 1.04, 95% CI = 0.96–1.12). Sex had a bivariate p-value of 0.544, which did not meet the p-value of less than or equal to 0.2 required for inclusion in the multivariable model (Table 2).

**Table 2:** Multivariable conditional logistic regression for determinants of TB disease development among children in central Ethiopia, 2022.

Cases	Controls		mCOR (95% CI)	p-value	mORadj (95% CI)	p-value
	Male	Female				
Male	56 (21.9%)	70 (27.3%)	1.11 (0.78–1.59)	0.544		
Female	63 (24.6%)	67 (26.2)	1.00			
	No BCG	BCG received				
No BCG	33 (12.9%)	66 (25.8%)	2.64 (1.67–4.18)	0.000*	2.11(1.28–3.48)	0.003**
BCG received	25 (9.8%)	132 (51.6%)	1.00		1.00	
	Household TB contact	No household TB contact				
Household TB contact	1 (0.4%)	42 (16.4%)	4.67 (2.27–9.59)	0.000*	4.28 (1.95–9.39)	0.000**
No household TB contact	9 (3.5%)	204 (79.7%)	1.00		1.00	
	Smoker family	No smoker family				
Smoker family	1 (0.4%)	21 (8.2%)	4.20 (1.58–11.14)	0.004*	3.15 (1.07–9.27)	0.037**
No smoker family	5 (2.0%)	229 (89.5%)	1.00		1.00	
	HIV infected	HIV uninfected or unknown				
HIV infected	1 (0.4%)	21 (8.2%)	10.50 (2.46–44.78)	0.001*	8.71 (1.96–38.66)	0.004**
HIV uninfected	2 (0.8%)	232 (90.6%)	1.00		1.00	
Household size			1.05(0.99–1.13)	0.118*	1.04 (0.96–1.12)	0.339

*Included in the multivariable model

**Statistical significant at p-value < 0.05

## Discussion

We confirmed that being unvaccinated for BCG, exposure to a household TB patient, exposure to a cigarette-smoking family member, and being HIV-infected were determinants of childhood TB disease development in central Ethiopia. This finding, therefore, will aid in the development of context-based intervention strategies to prevent and control TB [37], as well as serve as a foundation for future research on the subject.

Our study’s finding that BCG vaccination protects against TB disease is consistent with previous studies [18–20, 38, 39]. BCG vaccination activates CD4+ and CD8+ T lymphocytes, increasing IFN-γ production which enhances anti-mycobacterial action in macrophages [40].

Our finding is also consistent with prior studies that have found that close contact with a TB patient increases the risk of childhood TB [41–44], even regardless of whether the child was vaccinated for BCG at birth [14].

In line with prior individual studies [10, 45] and a meta-analysis [46], our study also found that living with a smoking family member is associated with TB disease development in children. The lack of an association between second-hand smoke exposure and TB in Taiwan’s study [47] could be attributed to the fact that its study participants did not have TB contact in their households, which could otherwise have an interaction effect with second-hand smoking.

HIV infection, a well-established risk factor for TB [4–6, 37], was also identified as one of the factors associated with TB development in children in our study, too, though the interval estimation was imprecise.

The lack of association with total household size in our study contradicts findings from studies in Thailand and Bangladesh [14, 43]. This disparity could be explained by the fact that our study measured household size, whereas those two studies measured the number of people living in a single room in a house.

We found no association between sex and TB in children. However, the debate over whether adult males are more likely than adult females to contract TB due to the confounding effect of factors such as smoking, alcohol, and drug use that expose them to TB or due to female hormones inhibiting TB disease development independently has continued [48].

In our result, the cases’ mean age (8.96 years) looked to be younger than the controls’ (10.3 years). The explanation for this is that the patients’ ages were recorded when they were diagnosed with TB, whereas the controls’ ages were obtained after the treatment outcome of the cases was determined and the matched control was chosen. This can be viewed as strength because it provided adequate time for clinical signs of potentially undetected TB in the controls, avoiding the bias of misclassifying the child with unrecognized TB as a control. This also allowed us to eliminate from our analysis any cases that were misdiagnosed as TB but later discontinued TB drugs due to a change in diagnosis [49].

The presence or absence of a BCG scar in the controls was determined during the face-to-face interview; however, identifying the presence of a BCG scars on the patients proved difficult. As a result, we based our analyses on orally reported BCG vaccination status rather than BCG scars. Oral reports of BCG vaccination status are reliable since the only vaccine provided via injection at birth or within two weeks of delivery in Ethiopia is the BCG vaccine [26].

One of our study’s limitations is that the HIV status of the majority (three-fourths) of the controls was unknown, as opposed to all the cases, which had known HIV status. In Ethiopia, HIV testing is required for TB patients [29]. We included HIV-unknown controls in the HIV-negative category in our analytic strategy. There could be HIV-positive children among the HIV-status unknown controls, causing the HIV-positive distribution among cases to be incorrectly larger than among controls, leading to an overestimation of a positive association between HIV infection and TB disease. However, HIV prevalence among persons aged 15 to 19 is as low as 0.1%. In such a case, the likelihood of having HIV-positive children among the 192 unknown status controls would be too small to influence our findings [50].

The other limitation of our study is that it did not look into whether undernutrition is a risk factor for TB or not because it was difficult to determine whether undernutrition or TB occurred first in TB patients. Even though diabetes or pre-diabetes increases the risk of TB [51], we left it out of the study because we were unable to collect diabetes or pre-diabetes data for both the cases and the controls. Another limitation of our study was that we ignored socioeconomic factors such as income level at the time of TB diagnosis since they were not feasible to quantify.

## Conclusions

No BCG vaccination at birth or within two weeks of birth was one of an independent determinant of TB disease development in children. With this finding, we recommend Ethiopia maintain its universal newborn BCG immunization strategy.

We also confirmed that the odds of developing TB disease were higher in children who were exposed to a pulmonary TB patient in their homes than those who were not exposed.

Children who lived in a household with a smoker had also more odds of developing TB than their peers who had never lived in a household with a smoker. This finding might be used to argue for the inclusion of an in-house smoking prohibition in houses with children in future national tobacco control legislation.

Even in this period of antiretroviral therapy, HIV infection remained a significant risk factor for the development of tuberculosis in children.

## Supporting information

S1 Dataset (DTA)
